# Circulating tumour cells and their association with bone metastases in patients with neuroendocrine tumours

**DOI:** 10.1038/s41416-018-0367-4

**Published:** 2019-01-14

**Authors:** Francesca M. Rizzo, Clare Vesely, Alexa Childs, Teresa Marafioti, Mohid S. Khan, Dalvinder Mandair, Mauro Cives, Leah Ensell, Helen Lowe, Ayse U. Akarca, TuVinh Luong, Martyn Caplin, Christos Toumpanakis, Daniel Krell, Christina Thirlwell, Franco Silvestris, John A. Hartley, Tim Meyer

**Affiliations:** 1 Department of Oncology, UCL Cancer Institute, University College London, London, UK; 2 Department of Pathology, UCL Cancer Institute, University College London, London, UK; 3 Wales Neuroendocrine Tumour Service, Department of Gastroenterology, University Hospital of Wales, Cardiff, UK; 4 Neuroendocrine Tumour Unit, Department of Gastroenterology, ENETS Centre of Excellence, Royal Free London NHS Foundation Trust, London, UK; 5 Department of Biomedical Sciences and Human Oncology, University of Bari “A. Moro”, Bari, Italy; 6 Department of Histopathology, Royal Free London NHS Foundation Trust, London, UK; 7 Neuroendocrine Tumour Unit, Department of Oncology, ENETS Centre of Excellence, Royal Free London NHS Foundation Trust, London, UK

**Keywords:** Bone metastases, Neuroendocrine cancer

## Abstract

**Background:**

Bone metastases are associated with a worse outcome in patients with neuroendocrine tumours (NETs). Tumour overexpression of C-X-C chemokine receptor 4 (CXCR4) appears predictive of skeletal involvement. We investigated the role of circulating tumour cells (CTCs) and CXCR4 expression on CTCs as potential predictors of skeleton invasion.

**Methods:**

Blood from patients with metastatic bronchial, midgut or pancreatic NET (pNET) was analysed by CellSearch. CXCR4 immunohistochemistry was performed on matched formalin-fixed paraffin-embedded (FFPE) samples.

**Results:**

Two hundred and fifty-four patients were recruited with 121 midgut and 119 pNETs, of which 51 and 36% had detectable CTCs, respectively. Bone metastases were reported in 30% of midgut and 23% of pNET patients and were significantly associated with CTC presence (*p* = 0.003 and *p* < 0.0001). In a subgroup of 40 patients, 85% patients with CTCs had CTCs positive for CXCR4 expression. The proportion of CXCR4-positive CTCs in patients with bone metastases was 56% compared to 35% in those without (*p* = 0.18) it. Staining for CXCR4 on matched FFPE tissue showed a trend towards a correlation with CXCR4 expression on CTCs (*p* = 0.08).

**Conclusions:**

CTC presence is associated with bone metastases in NETs. CXCR4 may be involved in CTC osteotropism and present a therapeutic target to reduce skeletal morbidity.

## Introduction

Neuroendocrine tumours (NETs) are a heterogeneous group of malignancies with diverse biological and clinical features. Although NETs may develop in almost all organs, they are prevalent within the lung, pancreas and gastrointestinal tract, and their incidence has markedly increased over the last four decades.^1^ The presence of bone metastases has been described in up to 32% of patients with NETs and is associated with a worse clinical outcome.^2,3^ The molecular mechanisms underlying the formation of skeletal metastases in NETs are not well understood, but the overexpression of C-X-C chemokine receptor type 4 (CXCR4) in primary tumours appears highly predictive of skeletal metastases.^4^

The CXCL12/CXCR4 axis is a critical molecular determinant in the bone homing of hematopoietic stem cells during foetal life and marrow transplantation.^5^ On the basis of the hematopoietic model, several authors have hypothesised that prostate and breast cancer cells may use a similar pathway to localise to the bone marrow.^6,7^ The functional role of the CXCL12/CXCR4 axis in NETs has recently been investigated. CXCR4 is overexpressed in gastro-entero-pancreatic and lung NETs where it signals through the mammalian target of rapamycin (mTOR) pathway inducing uncontrolled cell growth. Expression of CXCR4 is positively associated with a higher grade and poor patient outcome but has an inverse correlation with somatostatin receptor expression.^8–10^ Moreover, CXCR4 expression is upregulated by hypoxia, and its agonist stimulation activates the mitogen-activated protein kinase (MAPK) p42/44 signalling pathway, increasing ileal carcinoid cell migration.^11^ Novel findings on the functional role of the CXCL12/CXCR4 axis in modulating the osteotropism of NETs come from recent in vitro experimental models suggesting that CXCL12 conveys epithelial-mesenchymal transitions (EMT) promoting signals in NET cells through CXCR4, which in turn regulates transcriptional, morphological and functional modifications resulting in enhanced osteotropism of NET cells.^12^

The isolation and analysis of circulating tumour cells (CTCs) from the blood of patients with cancer offers the opportunity to investigate and understand the biology of metastatic process.

Using the CellSearch system we have previously demonstrated that CTCs are detectable in patients with NET and that their presence is an adverse prognostic factor.^13,14^ In addition, we have shown that early changes in CTC numbers predict survival in response to therapy^15^ and that therapeutic targets such as the somatostatin receptor can be detected on CTCs.^16^ A recent work suggests that CTC count can be used as an early predictor of bone metastatic potential in prostate,^17^ breast^18^ and lung cancer.^19^ In addition, the analysis of C-X-C chemokine receptor 4 (CXCR4) expression on CTCs is feasible and has been performed in small cell lung cancer patients in whom CXCR4 overexpression was shown to be predictive of shorter progression-free survival.^20^

Here we investigate the role of CTCs as a marker of bone metastases in a large cohort of NET patients and evaluate the expression of CXCR4 on CTCs as a potential predictor of skeletal invasion.

## Materials and methods

### CTC enumeration in NET patients

To be eligible for the study, patients had to be ≥ 18 years of age and had to have a histologically confirmed midgut, pancreatic or bronchial NET with metastatic disease. Patients who had undergone systemic anti-cancer therapy or embolization within the previous month were excluded while patients receiving long-term somatostatin analogues (SSAs) were included. Data were collected depending on age, gender, primary site, WHO grade (according to Ki67 proliferation index), metastatic sites and previous treatments. The study was approved by the Central Ethics Committee (IRAS ref. ^13^/LO/0376) and performed in accordance with the Declaration of Helsinki. All patients provided written informed consent. Patients provided 7.5 mL of blood samples, which were collected into CellSave tubes (Janssen Diagnostics, Raritan, NJ, USA), maintained at room temperature and processed within 96 h of collection. The CellSearch platform was used for the detection and enumeration of CTCs as previously described.^13^ Briefly, this semi-automated system enriches CTCs by EpCAM-targeted immunomagnetic selection, following which the CTCs are identified by positive immunofluorescent staining for pan-cytokeratin and DAPI, and negative staining for the leucocyte marker CD45.

### Detection of CXCR4 expression using CellSearch

**Fig. 1:**
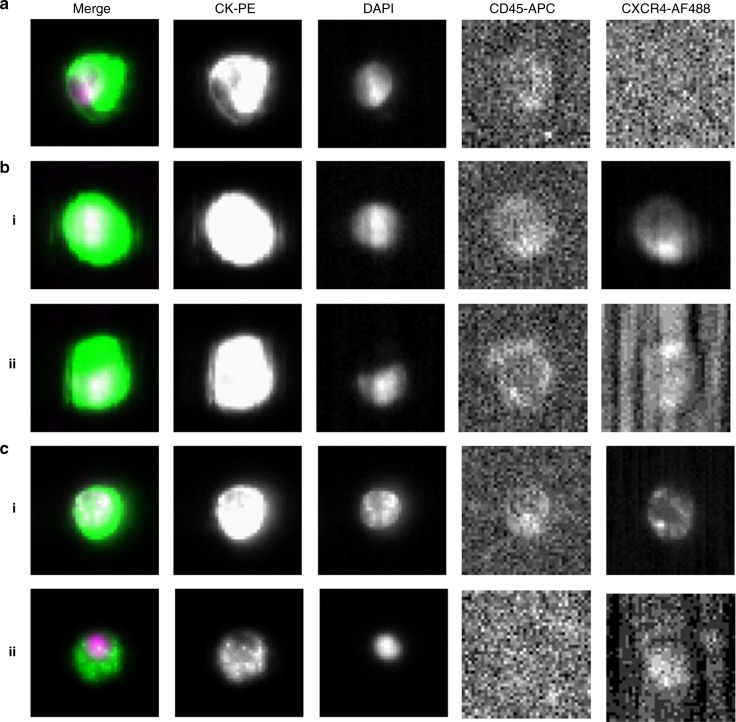
CellTracks Analyzer II example images. **a** Spiked BON1 cells, with no CXCR4 antibody used. **b** BON1-spiked blood samples run with CXCR4 antibody. i CXCR4-positive BON1 cell. ii CXCR4-negative BON1 cell. **c** Clinical validation in patient samples assayed with CXCR4 antibody. i CXCR4-positive CTC in patient ID1. ii CXCR4-negative CTC in patient ID1

In order to develop the assay for CXCR4 detection using the CellSearch platform, the pancreatic NET (pNET) cell line BON1 was used. This has previously been reported to express both EpCAM and CXCR4.^12^ Five hundred BON1 cells were spiked into 7.5 mL of healthy donor blood collected in CellSave preservative tubes and processed through the CellSearch platform. A fluorescein-conjugated antibody can be added for detection by the fourth fluorescence channel to further characterise the cells for an additional marker of interest. For this study, the AF488-conjugated anti-CXCR4 antibody [UMB2, Abcam, Ab208128] was used and cells were defined as positive for CXCR4 expression when staining was present in the fourth channel (Fig. 1a, b). Patient samples were analysed using the protocol optimised on cells (antibody concentration 1:10 and exposure time 5 s), and evaluations regarding the expression of CXCR4 on CTCs were made by two independent operators having no knowledge of the clinical status of the patients.

### Immunohistochemistry

Three-micron-thick formalin-fixed paraffin-embedded (FFPE) tissue sections of normal human tonsil, placenta and neuroendocrine tumours were subjected to conventional single immunohistochemistry (IHC) to study the expression of CXCR4 using a rabbit monoclonal anti-CXCR4 antibody [UMB2, Abcam, Ab124824]. Human tonsil and placenta tissues were used as the positive control tissue to define the optimal staining protocol, and a dilution of 1:50 was found to result in a specific staining pattern in the absence of a background signal. Parallel tests without the primary antibody served as the negative control. Immunostaining was performed using the automated BOND-III Autostainer (Leica Microsystems, UK) according to protocols described elsewhere.^21^ The semi-quantitative analysis of the stained sections was performed by an expert pathologist having no knowledge of the pathological data using a Nikon Eclipse E400 microscope. Immunoreactivity was quantified in terms of the percentage of positive tumour cells and staining intensity.

### Statistical analysis

Descriptive statistics was used for patient demographics as well as for clinical and pathological data. Receiver operating characteristic (ROC) curve analysis was used to set the optimal cut-off for CTCs to predict bone colonisation. The association between CTCs and clinicopathological features including grade and sites of metastases was evaluated by Fisher’s test and chi-square test, while the Spearman’s test was used to test the correlation between CXCR4 expression on CTCs and tumour tissue. A multivariable logistic regression model with backward stepwise elimination was used to adjust for confounders. All tests were two-sided, and statistical significance was declared at *p* ≤ 0.05. Statistical analysis was conducted using GraphPad Prism 5 software (GraphPad Software, L Jolla, CA, USA) and MedCalc 12.7 (MedCalc Software bvba, Ostend, Belgium).

## Results

Between 2009 and 2017, a total of 254 patients with metastatic NETs were recruited from the Royal Free Hospital, London, which include 119 patients with pNET, 121 patients with midgut NET and 14 patients with bronchial NET. Baseline characteristics are listed in Table 1. Pancreatic and midgut NETs represented the biggest subgroups (47 and 48% of patients, respectively). The majority of patients had grade-1 (39%) or -2 tumours (47%), with only 13% having grade-3 tumours. Of the 33 patients with G3 tumours, 20 patients had well-differentiated (WD) and 10 had poorly differentiated (PD) tumours. This information was not available for the remaining three patients. Almost all the patients had liver metastases (96%). Skeletal involvement was detected in 28% of patients and was more commonly observed in tumours arising from the lung (64%), compared with gastro-entero-pancreatic NETs (26%). At the time of enrolment, 27% of patients were treatment naive, 29% were receiving SSAs at the time of sampling and 43% had received alternative anti-cancer treatments in addition to, or instead of, SSAs. For the final group, the shortest time between the last treatment and CTC sampling was 1 month, with a median time of 13 months (ranging from 1 to 97 months).

**Table 1:** Clinical characteristics of NET patient samples

	All patients	Pancreatic	Midgut	Bronchial
	*(n* *=* *254)*	*(n* *=* *119)*	*(n* *=* *121)*	*(n* *=* *14)*
**Age at diagnosis (years)**				
Median	57	54	60.5	58.5
Range	24–81	24–79	27–81	40–76
**Sex**				
Male	137 (54%)	60 (50%)	69 (57%)	8 (57%)
Female	117 (46%)	59 (50%)	52 (43%)	6 (43%)
**Tumour grade**				
G1	99 (39%)	31 (26%)	66 (54%)	2 (14%)
G2	120 (47%)	65 (55%)	47 (39%)	8 (57%)
G3	33 (13%)	22 (18%)	7 (6%)	4 (29%)
Unknown	2 (1%)	1 (1%)	1 (1%)	0
**Metastatic sites**				
Liver	244 (96%)	118 (99%)	117 (97%)	9 (64%)
Lymph nodes	175 (69%)	68 (57%)	96 (79%)	11 (79%)
Bone	72 (28%)	27 (23%)	36 (30%)	9 (64%)
Lung	24 (9%)	6 (5%)	14 (11%)	4 (29%)
Peritoneum	83 (33%)	16 (13%)	65 (54%)	2 (14%)
**Previous treatments**				
Treatment naive	68 (27%)	35 (29%)	30 (25%)	3 (21%)
On SSAs	74 (29%)	21 (18%)	50 (41%)	3 (21%)
Previous anti-cancer treatments*	109 (43%)	62 (52%)	39 (32%)	8 (58%)
Unknown	3 (1%)	1 (1%)	2 (2%)	0
**CXCR4 subgroup analysis**				
**Primary site**	40	15 (38%)	11 (27%)	14 (35%)
**Age at diagnosis (years)**				
Median	57	52	59	59
Range	40–76	43–66	44–73	40–76
**Sex**				
Male	22 (55%)	9 (60%)	5 (45%)	8 (57%)
Female	18 (45%)	6 (40%)	6 (55%)	6 (43%)
**Tumour grade**				
G1	10 (25%)	2 (13%)	6 (55%)	2 (14%)
G2	22 (55%)	10 (67%)	4 (36%)	8 (57%)
G3	8 (20%)	3 (20%)	1 (9%)	4 (29%)
**Metastatic sites**				
Liver	33 (82%)	14 (93%)	10 (91%)	9 (64%)
Lymph nodes	27 (67%)	8 (53%)	8 (73%)	11 (79%)
Bone	20 (50%)	4 (27%)	7 (64%)	9 (64%)
Lung	5 (12%)	0	1 (9%)	4 (29%)
Peritoneum	7 (17%)	2 (13%)	3 (27%)	2 (14%)
**Previous treatments**				
Treatment naive	10 (25%)	6 (40%)	1 (9%)	3 (21%)
On SSAs	10 (25%)	1 (7%)	6 (55%)	3 (21%)
Previous anti-cancer treatments*	20 (50%)	8 (53%)	4 (36%)	8 (58%)

^*^Treatments include chemotherapy, radionuclide therapy, targeted therapy, interferon and liver-directed therapy.

*SSA* somatostatin analogue, *TAE* transarterial embolization

### CTC count in relation to bone colonisation

A CTC count of ≥ 1 was used for the initial exploratory analysis, since this threshold has been previously defined as the optimum threshold for prognostication.^14^ The association between CTC presence and grade, previous treatment and sites of metastases is shown in Table 2. Liver involvement has not been included in the correlation analysis, since 96% of the patients had liver metastases, and neither the bronchial NETs due to its low number. There was a significant association between CTC presence and grade in both pancreatic (*p* = 0.0193) and midgut (*p* = 0.0009) NETs, but there was no correlation between CTC presence and treatment in either pancreatic (*p* = 0.134) or midgut (*p* = 0.1418) NET. Of patients with metastatic pNET, 36% had detectable CTCs with a mean of 11 CTCs per 7.5 mL of blood (range 0–430, SD 53). Bone metastases were identified in 27 pNET patients (23%) by CT scan (59%), Ga68 PET-CT (41%), MRI (26%), Octreoscan (22%), FDG PET-CT (15%) and bone scan (11%). For 89% of patients imaging was performed within 3 months from enrolment. Applying the presence or absence of CTCs as a dichotomous variable, there was a significant association between bone metastases and CTC presence (*p* < 0.0001). There was no association between lung, peritoneal or lymph node metastases and CTC presence. To identify the optimal cut-off number of CTCs capable of predicting bone colonisation, ROC curve analysis was performed (Supplementary Figure S1).

**Table 2:** Association between CTC presence and grade, previous treatment and sites of metastases

	pNETs		*p*	Midgut NETs		*p*
	*(n* *=* *119)*			*(n* *=* *121)*		
	*CTC* *=* *0*	*CTC* *≥* *1*		*CTC* *=* *0*	*CTC* *≥* *1*	
**Tumour grade (%)**			0.0193			0.0009
G1	74	26		55	45	
G2	63	37		45	55	
G3	55	45		29	71	
**Previous treatment (*n*)**			0.134			0.1418
Treatment naive	23	12		19	11	
On SSAs	14	7		22	28	
Previous anti-cancer treatments*	39	23		16	23	
**Lung metastases (** ***n*** **)**			0.31			0.33
Yes	5	1		5	9	
No	71	42		54	53	
**Peritoneal metastases (** ***n*** **)**			0.12			0.80
Yes	13	3		31	34	
No	63	40		28	28	
**Bone metastases (** ***n*** **)**			< 0.0001			0.003
Yes	7	20		10	26	
No	69	23		49	36	
**Lymph node metastases (** ***n*** **)**			0.1861			0.4162
Yes	40	28		45	51	
No	36	15		14	11	

^*^Treatments include chemotherapy, radionuclide therapy, targeted therapy, interferon and liver-directed therapy.

In the pNET cohort, a CTC count of ≥ 2 distinguished between patients with and without bone metastases with a sensitivity of 66.7% (95% CI = 46–83.5%) and a specificity of 88% (95% CI = 79.6–93.9%), with an Area Under the Curve of 0.79 (95% CI = 0.7–0.86, *p* = 0.0001). Positive predictive value (PPV) and negative predictive value (NPV) for the defined cut-off were 62.1% (95% CI = 41.9–79.6%) and 90% (95% CI = 81.9–95.3%), respectively. By univariate analysis, a number of CTCs of ≥ 2 significantly predicted bone colonisation (*p* < 0.0001) (Supplementary Table S1). By logistic regression analysis, including the presence of at least two CTCs as a dependent variable and treatment, grade, lung, peritoneal, bone and lymph node metastases as independent variables, the association between bone metastases and CTCs was confirmed (*p* < 0.0001) and was not independent from grade.

Of patients with midgut tumours, 51% had detectable CTCs with a mean number of 15 (range 0–636, SD 66). Bone metastases were identified in 36 midgut patients (30%) by CT scan (44%), Ga68 PET-CT (42%), Octreoscan (42%), MRI (22%), FDG PET-CT (11%) and bone scan (3%). For 78% of patients imaging was performed within 3 months from enrolment. There was a significant association between bone metastases and CTC presence (*p* = 0.003). There was no association between lung, peritoneal or lymph node metastases and CTC presence. In the midgut cohort, the ROC curve analysis showed that a CTC count of ≥ 1 was able to distinguish between patients with and without bone metastases with a sensitivity of 72.2% (95% CI = 54.8–85.8%) and a specificity of 57.6% (95% CI = 46.4–68.3%), with an AUC of 0.65 (95% CI = 0.56–0.73, *p* = 0.0042) (Supplementary Figure 1B). PPV and NPV for the defined cut-off were 41.9% (95% CI = 29.5–55.2%) and 83.1% (95% CI = 71–91.6%), respectively. By logistic regression analysis, the association between bone metastases and CTC presence was confirmed (*p* = 0.002) and was independent from treatment, grade as well as from the other sites of metastases.

### CXCR4 detection in spiked cells and in CTCs from metastatic NET patients

Forty patients from Table 1, including 20 patients with bone metastases and 20 without bone metastatic disease, were further analysed for CXCR4 expression in CTCs (Table 1, CXCR4 subgroup analysis). As shown in Table 3, CTCs were detected in 20 (50%) patients including eight pancreatic, five bronchial and seven midgut NETs. Seventeen out of 20 patients with CTCs (85%) showed a subpopulation expressing CXCR4 (Fig. 1c), including 12 patients with bone metastases (92%) and 5 patients without bone metastases (71%) (*p* = 0.21). In those patients with CTC ≥ 1, the mean percentage of CXCR4^+^ CTCs was 56% for patients with bone metastases and 35% for patients without bone disease, respectively (*p* = 0.18).

**Table 3:** Characteristics of patients with detectable CTCs

ID	Primary site	Grade	Morphology	Ki67 (%)	Bone metastases	CTCs (*n*)	CXCR4^+^ CTCs (*n*)	CXCR4^+^ CTCs (%)	CXCR4^+^ tumour cells on FFPE (%)	Nuclear localisation (%)	Cytoplasmic localisation (%)
1	Pancreas	G2	WD	6%	Yes	361	141	39	100	100	0
2	Midgut	G2	WD	15%	Yes	18	6	33	100	40	60
3	Midgut	G1	WD	< 2%	Yes	8	4	50	90	0	90
4	Bronchial	G3	PD	50%	Yes	1	0	0	80	80	0
5	Bronchial	G2	WD	10–12%	Yes	179	113	63	100	100	0
6	Midgut	G2	WD	1–15%	Yes	2	2	100	ND	ND	ND
7	Bronchial	G2	WD	12%	Yes	6	3	50	60	50	10
8	Pancreas	G2	WD	5%	Yes	1	1	100	ND	ND	ND
9	Midgut	G1	WD	< 1%	Yes	23	21	91	ND	ND	ND
10	Bronchial	G2	WD	8%	Yes	78	8	10	20	5	15
11	Midgut	G1	WD	< 1%	Yes	3	2	67	100	100	0
12	Pancreas	G3	WD	50%	Yes	70	35	50	100	100	0
13	Pancreas	G2	WD	12%	Yes	4	3	75	100	100	0
14	Midgut	G3	PD	80%	No	23	7	30	ND	ND	ND
15	Pancreas	G2	WD	4%	No	1	0	0	100	100	0
16	Pancreas	G2	WD	15%	No	4	0	0	80	80	0
17	Pancreas	G2	WD	8%	No	1	1	100	ND	ND	ND
18	Pancreas	G2	WD	12%	No	28	4	14	40	40	0
19	Bronchial	G3	WD	25%	No	40	11	27	40	40	0
20	Midgut	G1	WD	< 2%	No	42	30	71	ND	ND	ND

*CTC* circulating tumour cells, *FFPE* formalin-fixed paraffin-embedded tissue, *WD* well-differentiated, *PD* poorly differentiated, *ND* not done

### Immunohistochemical analysis of CXCR4 expression

**Fig. 2:**
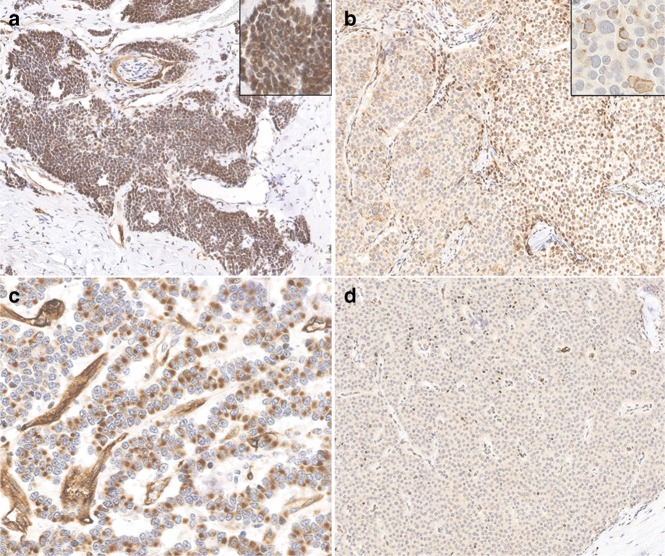
Immunohistochemistry for CXCR4 in NET patients. **a** Strong nuclear CXCR4 expression. **b** A heterogeneous case showing negative and nuclear positive cells, with some membrane and dot-like cytoplasmic staining indicated in the inset panel. **c** Prevalent dot-like cytoplasmic staining, with focal areas showing a weak cytoplasmic staining. **d** CXCR4-negative staining

**Fig. 3:**
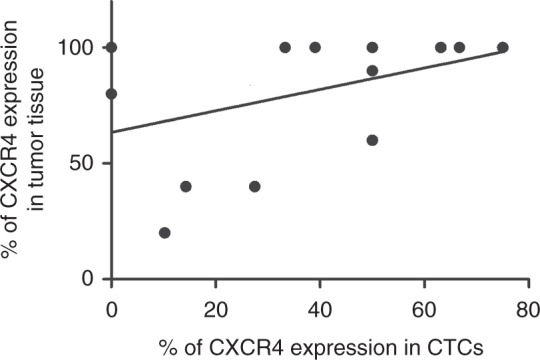
Correlation plot of percentage of CXCR4 expression in CTCs versus percentage of CXCR4 expression in matched FFPE samples

Of the 20 patients with detectable CTCs, 14 had blocks of FFPE tissue available for a further IHC analysis. Staining for CXCR4 was predominantly nuclear with moderate to strong intensity (Fig. 2a). Interestingly, patients with G3 and PD tumours had nuclear CXCR4 staining only. One patient with a WD G1 midgut tumour had only cytoplasmic staining while some cases were heterogeneous with both negative and positive cells with nuclear and cytoplasmic (dot-like or granular) staining (Fig. 2b, c). Figure 2d shows a CXCR4-negative sample from a patient with no detectable CTCs, therefore not included in Table 3. There was a trend towards a significant correlation between CXCR4 expression in tumour tissue and CTCs (*r*_s_ = 0.48, 95% CI = -0.08, 0.81; *p* = 0.08, Fig. 3).

## Discussion

Bone metastases have been reported in up to 32% of NET patients^2^ and are a negative prognostic factor associated with a median survival of less than 3 years from the diagnosis of bone lesions.^3^ Hence, osteotropic NETs may be regarded as an aggressive subtype, and an understanding of the molecular mechanisms of osteotropism may inform the development of therapeutic interventions to reduce skeletal morbidity. In this study, we investigated the correlation between CTC count and bone involvement in metastatic NETs and explored the role of the CXCL12/CXCR4 axis in the biology of bone-colonising NETs.

In keeping with previously published data,^14,16^ CTCs were isolated in 43% of metastatic NET patients overall, with a higher proportion of midgut patients having detectable CTCs compared with pancreatic patients. A significant association was found between CTC presence and grade, while previous treatments did not affect CTC count in both pancreatic and midgut NETs. We showed that CTC presence significantly correlates with bone involvement while there is no association with other metastatic sites of the disease including lung, peritoneum and lymph nodes. The high PPV and NPV, particularly for pNETs, suggest that CTCs could be a predictive marker of bone metastases. Cancer cells are able to remain as ‘niche-engaged’ dormant cells in the bone marrow for years before switching to a proliferative phenotype and eventually causing overt metastases.^22^ Therefore, CTCs may be identified in those patients with subclinical bone metastases before they become clinically or radiologically manifest. The association between CTC count by CellSearch and bone metastases has also been reported in patients with prostate,^17^ breast^18^ and lung cancer^19^ although the molecular mechanism has not been explored.

Here we also investigate potential molecular mechanisms of bone metastasis in CTCs. The CXCL12/CXCR4 axis is a physiological mediator of bone marrow homing and quiescence of hematopoietic stem cells^5^ and also regulates the migration of breast and prostate cancer cells towards tissues with a high-CXCL12 expression including bone.^6,7^ Based on previous in vitro data showing that the CXCL12/CXCR4 axis regulates the osteotropism of NET cancer cells,^12^ we analysed the expression of CXCR4 on CTCs in a proportion of our patients and found that it tended to be higher in patients with bone metastases than in those without it. Our results did not reach statistical significance, likely due to the small number of patients included in this subgroup analysis, but the limited sensitivity of imaging to detect subclinical bone metastases may be another important factor.

Immunohistochemical analysis of CXCR4 expression was in line with previous studies showing the association between subcellular localisation and advanced and PD tumours,^23–25^ suggesting a possible involvement of CXCR4 in the intracellular signalling of NET cells as a functional nuclear importable receptor, which requires further investigation at the molecular level. Some degree of discordance was described between CXCR4 expression on CTCs and matched FFPE samples. A positive CXCR4 expression was detected by IHC in three cases that were negative in CTCs, despite the fact that the same antibody clone was used. There are a number of explanations that may account for these discrepancies. First, and most likely, this difference can be explained by sampling bias as very small numbers of CTCs, ranging from 1 to 4, were found in the three discordant cases. A second possible reason for discrepancy, which may be relevant for one of the three discordant cases, could be that the histology specimen was obtained 3 years before the CTC sample was taken, and the reduced CXCR4 expression seen in CTCs might have arisen from tumour evolution. It has been reported that CXCR4 expression can be dynamically modulated over the course of the disease in response to changes in the microenvironment, especially in the process of tumour-stromal interactions.^26^ A final explanation is the heterogeneity of expression within tumours and between CTCs, clearly seen in some of our samples. Our group has already reported heterogeneity and discordance between biomarker expression on CTCs and archived tissue as a result of phenotype changes during CTC translocation from tissue to blood.^16^

A potential limitation of the study is that the detection of CTCs was based on EpCAM expression, and this may have missed subpopulations of EpCAM-negative cells, such as those undergoing EMT. To date, there are no studies demonstrating that EMT occurs in NET, and the relevance of this putative population is unclear. Furthermore, recent studies indicate that tumour cell lines that are arrested in a mesenchymal state by the expression of EMT-inducing proteins are unable to form overt metastases after homing in distant organs.^27,28^ On the contrary, cells with an intermediate (epithelial and mesenchymal) phenotype, therefore still detected by the CellSearch system, have the highest plasticity and metastatic potential,^29^ thus confirming the importance of detecting this subgroup of cells.

In conclusion, we have shown a correlation between CTCs detected by CellSearch and bone metastases, which may inform clinical investigations and surveillance strategies. Additionally, our findings suggest a potential role for CXCR4, which may have important implications for therapy since CXCR4-directed imaging modalities^30^ as well as inhibitors and monoclonal antibodies^8,31^ are available and are currently being evaluated in NETs.

## Supplementary information

Figure S1

Table S1

## Availability of data and material

The datasets generated during and/or analysed during the current study are available from the corresponding author on reasonable request.
