# Supplementary figures and images for: Circulating tumour cells and their association with bone metastases in patients with neuroendocrine tumours
Source: Br J Cancer. 2019 Jan 14;120(3):294–300. doi: 10.1038/s41416-018-0367-4 (PMC6353867; doi:10.1038/s41416-018-0367-4)

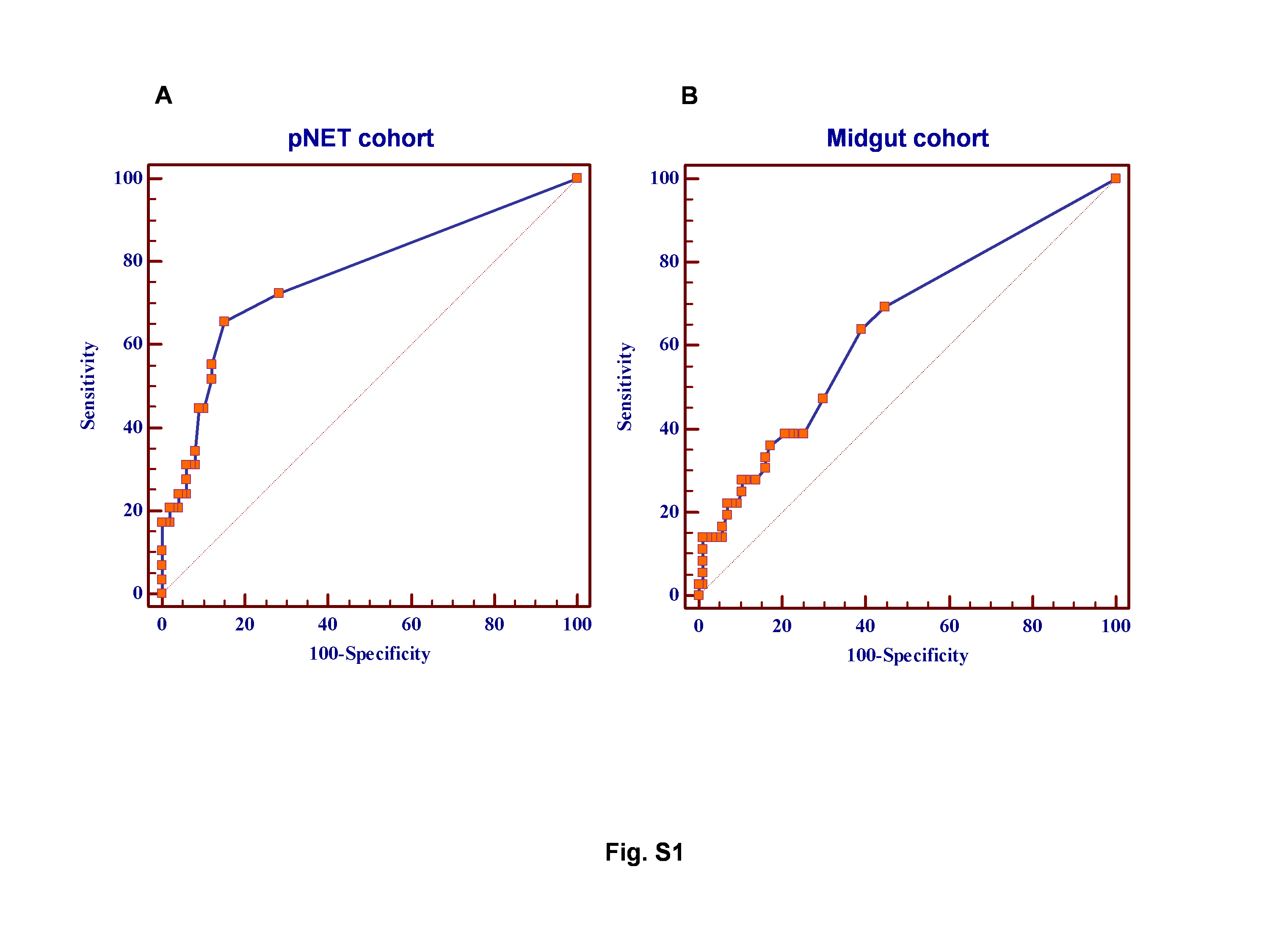

Supplement: Supplementary file 1 — Figure S1 [file 41416_2018_367_MOESM1_ESM.tif]
