# Supplementary material for: Circulating tumour cells and their association with bone metastases in patients with neuroendocrine tumours
Source: Br J Cancer. 2019 Jan 14;120(3):294–300. doi: 10.1038/s41416-018-0367-4 (PMC6353867; doi:10.1038/s41416-018-0367-4)
Supplement: Supplementary file 2 — Table S1 [file 41416_2018_367_MOESM2_ESM.doc]

**Supplementary Table 1.** Association between CTC count of ≥2 and grade, previous treatment and sites of metastases.

|  | **pNETs (*n*=119)** | | ***p*** |
| --- | --- | --- | --- |
|  | **CTC≤1** | **CTC>1** |  |
| **Tumor grade (%)**  **G1**  **G2**  **G3** | 74  63  55 | 26  37  45 | <0.00013 |
| **Previous treatment (n)**  **Treatment naïve**  **On SSAs**  **Previous anti-cancer treatments*** | 28  17  45 | 7  4  17 | 0.61 |
| **Lung metastases (n)**  **Yes**  **No** | 5  85 | 1  28 | 0.652 |
| **Peritoneal metastases (n)**  **Yes**  **No** | 15  75 | 1  28 | 0.0696 |
| **Bone metastases (n)**  **Yes**  **No** | 9  81 | 18  11 | <0.0001 |
| **Lymph node metastases (n)**  **Yes**  **No** | 49  41 | 19  10 | 0.2947 |

*Treatments include chemotherapy, radionuclide therapy, targeted therapy, interferon and liver-directed therapy.
